# 4,11-Diaza-1,8-diazo­niacyclo­tetra­decane dichloride hemihydrate

**DOI:** 10.1107/S1600536809031110

**Published:** 2009-08-12

**Authors:** Nam-Ho Kim, In-Chul Hwang, Kwang Ha

**Affiliations:** a School of Applied Chemical Engineering, the Research Institute of Catalysis, Chonnam National University, Gwangju 500-757, Republic of Korea; b Institute of Basic Sciences, Pohang University of Science and Technology, Pohang 790-784, Republic of Korea

## Abstract

In the title compound, C_10_H_26_N_4_^2+^·2Cl^−^·0.5H_2_O, the cyclam (1,4,8,11-tetra­azacyclo­tetra­decane) dication adopts an endodentate conformation which my be inflenced by intra­molecular N—H⋯N hydrogen bonding. In the crystal structure, the components are linked by N—H⋯Cl and O—H⋯Cl hydrogen bonds into chains along [100]. The water molecule is disordered over two sites in a 50:50 ratio.

## Related literature

For the crystal structure of [H_2_(cyclam)](ClO_4_)_2_, see: Nave & Truter (1974 ▶). For the crystal structures of [H_4_(cyclam)]*X·n*H_2_O [*X* = Cl_4_, Br_4_, (ClO_4_)_4_, (SCN)_4_, (SO_4_)_2_ or (*p*-CH_3_C_6_H_4_SO_3_)_4_], see: Robinson *et al.* (1989 ▶); Subramanian & Zaworotko (1995 ▶).
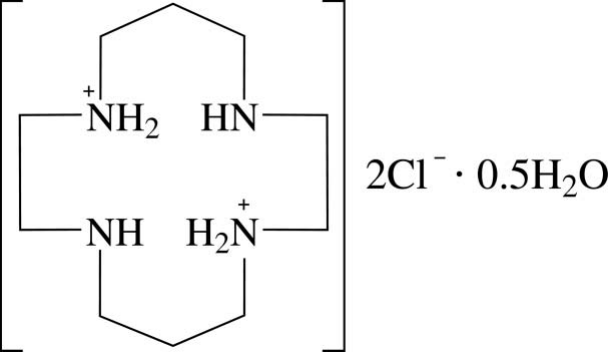


## Experimental

### 

#### Crystal data

C_10_H_26_N_4_^2+^·2Cl^−^·0.5H_2_O
*M* *_r_* = 282.25
Monoclinic, 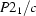

*a* = 6.827 (7) Å
*b* = 14.071 (16) Å
*c* = 16.055 (16) Å
β = 97.84 (3)°
*V* = 1528 (3) Å^3^
*Z* = 4
Mo *K*α radiation
μ = 0.41 mm^−1^
*T* = 293 K
0.25 × 0.10 × 0.10 mm

#### Data collection

Bruker SMART 1000 CCD diffractometer
Absorption correction: multi-scan (*SADABS*; Bruker, 2000 ▶) *T*_min_ = 0.806, *T*_max_ = 0.959
8725 measured reflections
3136 independent reflections
1280 reflections with *I* > 2σ(*I*)
*R*_int_ = 0.110

#### Refinement

*R*[*F* ^2^ > 2σ(*F* ^2^)] = 0.072
*wR*(*F* ^2^) = 0.151
*S* = 0.98
3136 reflections
154 parameters
H-atom parameters constrained
Δρ_max_ = 0.23 e Å^−3^
Δρ_min_ = −0.22 e Å^−3^

### 

Data collection: *SMART* (Bruker, 2000 ▶); cell refinement: *SAINT* (Bruker, 2000 ▶); data reduction: *SAINT*; program(s) used to solve structure: *SHELXS97* (Sheldrick, 2008 ▶); program(s) used to refine structure: *SHELXL97* (Sheldrick, 2008 ▶); molecular graphics: *ORTEP-3* (Farrugia, 1997 ▶) and *PLATON* (Spek, 2009 ▶); software used to prepare material for publication: *SHELXL97*.

## Supplementary Material

Crystal structure: contains datablocks global, I. DOI: 10.1107/S1600536809031110/lh2872sup1.cif

Structure factors: contains datablocks I. DOI: 10.1107/S1600536809031110/lh2872Isup2.hkl

Additional supplementary materials:  crystallographic information; 3D view; checkCIF report

## Figures and Tables

**Table 1:** Hydrogen-bond geometry (Å, °)

*D*—H⋯*A*	*D*—H	H⋯*A*	*D*⋯*A*	*D*—H⋯*A*
N1—H11⋯Cl2	0.86	2.67	3.356 (5)	138
N2—H21⋯Cl1	0.86	2.29	3.077 (5)	153
N2—H22⋯N1	0.86	2.29	2.882 (6)	126
N2—H22⋯N3	0.86	2.37	2.890 (5)	119
N3—H31⋯Cl1^i^	0.86	2.61	3.340 (4)	143
N4—H41⋯N1	0.86	2.40	2.899 (5)	118
N4—H41⋯N3	0.86	2.28	2.882 (6)	127
N4—H42⋯Cl2^i^	0.86	2.38	3.122 (5)	144
O1—H1*O*⋯Cl2^ii^	0.83	2.35	3.175 (8)	175
O1—H2*O*⋯Cl2^iii^	0.83	2.34	3.160 (8)	175

Symmetry codes: (i) 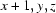; (ii) 
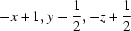
; (iii) 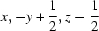.

**Fig. 1.**
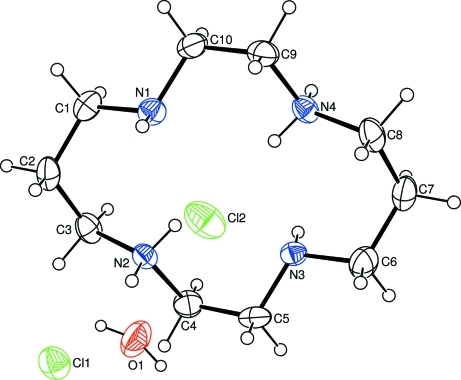
The molecular structure of the title compound, with displacement ellipsoids drawn at the 30% probability level for non-H atoms.

**Fig. 2.**
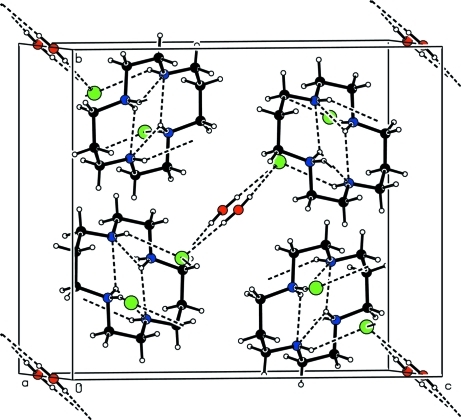
Part of the crystal structure with hydrogen bonds shown as dashed lines.

## supplementary crystallographic information

### Comment

The asymmetric unit of the title compound, C_10_H_26_N_4_^2+^.2Cl^-^.0.5H_2_O, consists of a doubly protonated 1,4,8,11-tetraazacyclotetradecane (cyclam) dication, two chloride anions and one half of a solvent water molecule (Fig. 1). The macrocyclic dication contains two protonated N atoms and two secondary amine N atoms, and is in an endodentate conformation with the N atoms oriented towards the centre of the macrocyclic cavity. The conformation of the dication may be influnced by intramolecular N—H···N hydrogen bonding (Table 1 and Fig. 2). The N2—C4—C5—N3 and N4—C9—C10—N1 torsion angles [-62.4 (5)° and 62.1 (5)°, respectively] display the *gauche* conformation for these two groups within the dication. A similar conformation is also observed in the structures cyclam (Robinson *et al.*, 1989) and [H_2_(cyclam)](ClO_4_)_2_ (Nave & Truter, 1974). Unlike cyclam and the dication, the tetracation, [H_4_(cyclam)]^4+^, adopts an exodentate conformation, in which all four N atoms are oriented away from the ring cavity, occupying positions as far away as possible from each other on the ring periphery (Robinson *et al.*, 1989; Subramanian & Zaworotko, 1995). The components of the crystal structure are linked by N—H···Cl and O—H···Cl hydrogen bonds into one-dimensional chains along [100] (Table 1 and Fig. 2).

### Experimental

Single crystals of the title compound were unexpectedly obtained as a byproduct of an attempted preparation of a Pd(II) complex by reacting Na_2_PdCl_4_ (0.073 g, 0.25 mmol) and 1,4,8,11-tetraazacyclotetradecane (0.100 g, 0.50 mmol) in H_2_O (10 ml) under reflux for 2 h. Crystals suitable for X-ray analysis were obtained by slow evaporation of a CH_2_Cl_2_ solution of the white reaction product.

### Refinement

H atoms were positioned geometrically and allowed to ride on their respective carrier atoms [C—H = 0.97 Å, N—H = 0.86 Å, O—H = 0.83 Å and *U*_iso_(H) = 1.2*U*_eq_(C, N) or 1.5*U*_eq_(O)].

### Crystal data

**Table:**

C_10_H_26_N_4_^2+^·2Cl^−^·0.5H_2_O	*F*(000) = 612
*M**_r_* = 282.25	*D*_x_ = 1.227 Mg m^−^^3^
Monoclinic, *P*2_1_/*c*	Mo *K*α radiation, λ = 0.71073 Å
Hall symbol: -P 2ybc	Cell parameters from 418 reflections
*a* = 6.827 (7) Å	θ = 2.6–16.2°
*b* = 14.071 (16) Å	µ = 0.41 mm^−^^1^
*c* = 16.055 (16) Å	*T* = 293 K
β = 97.84 (3)°	Needle, colorless
*V* = 1528 (3) Å^3^	0.25 × 0.10 × 0.10 mm
*Z* = 4	

### Data collection

**Table:**

Bruker SMART 1000 CCD diffractometer	3136 independent reflections
Radiation source: fine-focus sealed tube	1280 reflections with *I* > 2σ(*I*)
graphite	*R*_int_ = 0.110
φ and ω scans	θ_max_ = 26.4°, θ_min_ = 2.6°
Absorption correction: multi-scan (*SADABS*; Bruker, 2000)	*h* = −5→8
*T*_min_ = 0.806, *T*_max_ = 0.959	*k* = −17→17
8725 measured reflections	*l* = −18→20

### Refinement

**Table:**

Refinement on *F*^2^	Primary atom site location: structure-invariant direct methods
Least-squares matrix: full	Secondary atom site location: difference Fourier map
*R*[*F*^2^ > 2σ(*F*^2^)] = 0.072	Hydrogen site location: inferred from neighbouring sites
*wR*(*F*^2^) = 0.151	H-atom parameters constrained
*S* = 0.98	*w* = 1/[σ^2^(*F*_o_^2^) + (0.0321*P*)^2^] where *P* = (*F*_o_^2^ + 2*F*_c_^2^)/3
3136 reflections	(Δ/σ)_max_ < 0.001
154 parameters	Δρ_max_ = 0.23 e Å^−^^3^
0 restraints	Δρ_min_ = −0.22 e Å^−^^3^

### Special details

**Table:**

Geometry. All e.s.d.'s (except the e.s.d. in the dihedral angle between two l.s. planes) are estimated using the full covariance matrix. The cell e.s.d.'s are taken into account individually in the estimation of e.s.d.'s in distances, angles and torsion angles; correlations between e.s.d.'s in cell parameters are only used when they are defined by crystal symmetry. An approximate (isotropic) treatment of cell e.s.d.'s is used for estimating e.s.d.'s involving l.s. planes.
Refinement. Refinement of *F*^2^ against ALL reflections. The weighted *R*-factor *wR* and goodness of fit *S* are based on *F*^2^, conventional *R*-factors *R* are based on *F*, with *F* set to zero for negative *F*^2^. The threshold expression of *F*^2^ > σ(*F*^2^) is used only for calculating *R*-factors(gt) *etc*. and is not relevant to the choice of reflections for refinement. *R*-factors based on *F*^2^ are statistically about twice as large as those based on *F*, and *R*- factors based on ALL data will be even larger.

### Fractional atomic coordinates and isotropic or equivalent isotropic displacement parameters (Å^2^)

**Table:**

	*x*	*y*	*z*	*U*_iso_*/*U*_eq_	Occ. (<1)
N1	0.7249 (6)	0.4173 (2)	0.2123 (2)	0.0456 (11)	
H11	0.6227	0.4290	0.2365	0.055*	
N2	0.5366 (5)	0.2363 (2)	0.1733 (2)	0.0418 (10)	
H21	0.4233	0.2397	0.1909	0.050*	
H22	0.6292	0.2611	0.2084	0.050*	
N3	0.8016 (5)	0.1650 (2)	0.3153 (2)	0.0414 (10)	
H31	0.9071	0.1604	0.2918	0.050*	
N4	0.9882 (5)	0.3461 (3)	0.3557 (2)	0.0434 (10)	
H41	0.9066	0.3187	0.3175	0.052*	
H42	1.0960	0.3410	0.3339	0.052*	
C1	0.6795 (8)	0.4357 (3)	0.1219 (3)	0.0552 (15)	
H1A	0.7938	0.4201	0.0947	0.066*	
H1B	0.6515	0.5028	0.1129	0.066*	
C2	0.5032 (7)	0.3779 (4)	0.0822 (3)	0.0567 (15)	
H2A	0.3902	0.3928	0.1105	0.068*	
H2B	0.4709	0.3964	0.0238	0.068*	
C3	0.5392 (7)	0.2717 (4)	0.0867 (3)	0.0526 (14)	
H3A	0.4377	0.2395	0.0488	0.063*	
H3B	0.6662	0.2575	0.0689	0.063*	
C4	0.5882 (7)	0.1337 (3)	0.1843 (3)	0.0531 (14)	
H4A	0.7030	0.1196	0.1570	0.064*	
H4B	0.4789	0.0950	0.1585	0.064*	
C5	0.6317 (7)	0.1110 (3)	0.2767 (3)	0.0535 (14)	
H5A	0.5175	0.1263	0.3040	0.064*	
H5B	0.6582	0.0436	0.2840	0.064*	
C6	0.8527 (8)	0.1452 (4)	0.4051 (3)	0.0612 (15)	
H6A	0.8830	0.0782	0.4127	0.073*	
H6B	0.7395	0.1592	0.4335	0.073*	
C7	1.0288 (8)	0.2034 (4)	0.4450 (3)	0.0596 (15)	
H7A	1.0624	0.1839	0.5031	0.071*	
H7B	1.1414	0.1895	0.4161	0.071*	
C8	0.9932 (8)	0.3095 (4)	0.4424 (3)	0.0559 (14)	
H8A	0.8686	0.3234	0.4625	0.067*	
H8B	1.0977	0.3412	0.4792	0.067*	
C9	0.9384 (7)	0.4482 (3)	0.3442 (3)	0.0520 (14)	
H9A	1.0482	0.4867	0.3699	0.062*	
H9B	0.8237	0.4630	0.3714	0.062*	
C10	0.8954 (7)	0.4709 (3)	0.2517 (3)	0.0513 (14)	
H10A	0.8697	0.5384	0.2444	0.062*	
H10B	1.0099	0.4553	0.2246	0.062*	
Cl1	0.08543 (19)	0.22997 (10)	0.17133 (7)	0.0588 (4)	
Cl2	0.4464 (2)	0.35637 (10)	0.36069 (8)	0.0720 (5)	
O1	0.6491 (12)	0.0033 (6)	0.0003 (5)	0.105 (3)	0.50
H1O	0.6316	−0.0355	0.0371	0.157*	0.50
H2O	0.6030	0.0408	−0.0370	0.157*	0.50

### Atomic displacement parameters (Å^2^)

**Table:**

	*U*^11^	*U*^22^	*U*^33^	*U*^12^	*U*^13^	*U*^23^
N1	0.042 (2)	0.049 (3)	0.047 (3)	−0.002 (2)	0.012 (2)	0.002 (2)
N2	0.039 (2)	0.047 (3)	0.039 (2)	−0.004 (2)	0.002 (2)	−0.0032 (19)
N3	0.039 (2)	0.043 (3)	0.044 (3)	−0.004 (2)	0.011 (2)	0.0023 (19)
N4	0.041 (2)	0.054 (3)	0.035 (2)	−0.005 (2)	0.005 (2)	−0.0072 (19)
C1	0.065 (4)	0.051 (4)	0.050 (4)	0.004 (3)	0.012 (3)	0.012 (3)
C2	0.053 (4)	0.074 (4)	0.041 (3)	0.010 (3)	−0.002 (3)	0.011 (3)
C3	0.049 (3)	0.073 (4)	0.036 (3)	−0.002 (3)	0.004 (3)	−0.006 (3)
C4	0.059 (4)	0.043 (4)	0.057 (4)	0.000 (3)	0.006 (3)	−0.007 (3)
C5	0.051 (3)	0.039 (3)	0.073 (4)	−0.013 (3)	0.016 (3)	0.001 (3)
C6	0.064 (4)	0.072 (4)	0.049 (4)	−0.002 (3)	0.012 (3)	0.012 (3)
C7	0.061 (4)	0.075 (4)	0.041 (3)	0.008 (3)	0.003 (3)	0.015 (3)
C8	0.055 (4)	0.076 (4)	0.034 (3)	−0.001 (3)	−0.001 (3)	−0.003 (3)
C9	0.047 (3)	0.041 (3)	0.066 (4)	−0.005 (3)	0.004 (3)	−0.007 (3)
C10	0.049 (3)	0.039 (3)	0.067 (4)	−0.003 (3)	0.012 (3)	0.009 (3)
Cl1	0.0451 (8)	0.0785 (10)	0.0542 (9)	−0.0012 (7)	0.0115 (7)	0.0022 (7)
Cl2	0.0491 (9)	0.0979 (12)	0.0713 (10)	−0.0110 (8)	0.0162 (8)	−0.0200 (8)
O1	0.111 (7)	0.116 (7)	0.088 (6)	0.008 (6)	0.020 (6)	0.047 (5)

### Geometric parameters (Å, °)

**Table:**

N1—C10	1.457 (6)	C3—H3B	0.9700
N1—C1	1.466 (5)	C4—C5	1.508 (6)
N1—H11	0.8600	C4—H4A	0.9700
N2—C3	1.479 (5)	C4—H4B	0.9700
N2—C4	1.491 (5)	C5—H5A	0.9700
N2—H21	0.8600	C5—H5B	0.9700
N2—H22	0.8600	C6—C7	1.522 (6)
N3—C5	1.453 (5)	C6—H6A	0.9700
N3—C6	1.463 (5)	C6—H6B	0.9700
N3—H31	0.8601	C7—C8	1.512 (6)
N4—C8	1.480 (5)	C7—H7A	0.9700
N4—C9	1.483 (5)	C7—H7B	0.9700
N4—H41	0.8600	C8—H8A	0.9700
N4—H42	0.8600	C8—H8B	0.9700
C1—C2	1.519 (6)	C9—C10	1.507 (6)
C1—H1A	0.9700	C9—H9A	0.9700
C1—H1B	0.9700	C9—H9B	0.9700
C2—C3	1.515 (6)	C10—H10A	0.9700
C2—H2A	0.9700	C10—H10B	0.9700
C2—H2B	0.9700	O1—H1O	0.8253
C3—H3A	0.9700	O1—H2O	0.8266
			
C10—N1—C1	112.8 (4)	C5—C4—H4B	109.8
C10—N1—H11	110.7	H4A—C4—H4B	108.2
C1—N1—H11	109.8	N3—C5—C4	110.3 (4)
C3—N2—C4	113.8 (3)	N3—C5—H5A	109.6
C3—N2—H21	114.6	C4—C5—H5A	109.6
C4—N2—H21	102.9	N3—C5—H5B	109.6
C3—N2—H22	112.1	C4—C5—H5B	109.6
C4—N2—H22	100.0	H5A—C5—H5B	108.1
H21—N2—H22	112.2	N3—C6—C7	112.4 (4)
C5—N3—C6	112.9 (4)	N3—C6—H6A	109.1
C5—N3—H31	116.2	C7—C6—H6A	109.1
C6—N3—H31	108.4	N3—C6—H6B	109.1
C8—N4—C9	115.4 (3)	C7—C6—H6B	109.1
C8—N4—H41	116.4	H6A—C6—H6B	107.9
C9—N4—H41	103.3	C8—C7—C6	113.9 (4)
C8—N4—H42	116.2	C8—C7—H7A	108.8
C9—N4—H42	103.0	C6—C7—H7A	108.8
H41—N4—H42	100.4	C8—C7—H7B	108.8
N1—C1—C2	111.6 (4)	C6—C7—H7B	108.8
N1—C1—H1A	109.3	H7A—C7—H7B	107.7
C2—C1—H1A	109.3	N4—C8—C7	110.6 (4)
N1—C1—H1B	109.3	N4—C8—H8A	109.5
C2—C1—H1B	109.3	C7—C8—H8A	109.5
H1A—C1—H1B	108.0	N4—C8—H8B	109.5
C3—C2—C1	113.2 (4)	C7—C8—H8B	109.5
C3—C2—H2A	108.9	H8A—C8—H8B	108.1
C1—C2—H2A	108.9	N4—C9—C10	109.8 (4)
C3—C2—H2B	108.9	N4—C9—H9A	109.7
C1—C2—H2B	108.9	C10—C9—H9A	109.7
H2A—C2—H2B	107.7	N4—C9—H9B	109.7
N2—C3—C2	110.7 (4)	C10—C9—H9B	109.7
N2—C3—H3A	109.5	H9A—C9—H9B	108.2
C2—C3—H3A	109.5	N1—C10—C9	110.8 (4)
N2—C3—H3B	109.5	N1—C10—H10A	109.5
C2—C3—H3B	109.5	C9—C10—H10A	109.5
H3A—C3—H3B	108.1	N1—C10—H10B	109.5
N2—C4—C5	109.5 (4)	C9—C10—H10B	109.5
N2—C4—H4A	109.8	H10A—C10—H10B	108.1
C5—C4—H4A	109.8	H1O—O1—H2O	149.6
N2—C4—H4B	109.8		
			
C10—N1—C1—C2	179.2 (4)	C5—N3—C6—C7	−179.1 (4)
N1—C1—C2—C3	−63.7 (5)	N3—C6—C7—C8	62.9 (6)
C4—N2—C3—C2	−175.0 (4)	C9—N4—C8—C7	175.4 (4)
C1—C2—C3—N2	73.6 (5)	C6—C7—C8—N4	−71.8 (5)
C3—N2—C4—C5	165.8 (4)	C8—N4—C9—C10	−167.9 (4)
C6—N3—C5—C4	−179.4 (4)	C1—N1—C10—C9	−179.0 (4)
N2—C4—C5—N3	−62.4 (5)	N4—C9—C10—N1	62.1 (5)

### Hydrogen-bond geometry (Å, °)

**Table:**

*D*—H···*A*	*D*—H	H···*A*	*D*···*A*	*D*—H···*A*
N1—H11···Cl2	0.86	2.67	3.356 (5)	138
N2—H21···Cl1	0.86	2.29	3.077 (5)	153
N2—H22···N1	0.86	2.29	2.882 (6)	126
N2—H22···N3	0.86	2.37	2.890 (5)	119
N3—H31···Cl1^i^	0.86	2.61	3.340 (4)	143
N4—H41···N1	0.86	2.40	2.899 (5)	118
N4—H41···N3	0.86	2.28	2.882 (6)	127
N4—H42···Cl2^i^	0.86	2.38	3.122 (5)	144
O1—H1O···Cl2^ii^	0.83	2.35	3.175 (8)	175
O1—H2O···Cl2^iii^	0.83	2.34	3.160 (8)	175

Symmetry codes: (i) *x*+1, *y*, *z*; (ii) −*x*+1, *y*−1/2, −*z*+1/2; (iii) *x*, −*y*+1/2, *z*−1/2.
